# Phenothiazine-linked glutamic acid dendrons: an easy access and a new class of SARS-CoV-2 main protease inhibitors

**DOI:** 10.1098/rsos.241628

**Published:** 2025-04-02

**Authors:** Sameer Singh, Aditi Gangopadhyay, Sriram D, Manab Chakravarty

**Affiliations:** ^1^ Department of Chemistry, Birla Institute of Technology & Science Pilani - Hyderabad Campus, Hyderabad, Telangana, India; ^2^ Department of Chemical Technology, University of Calcutta, Kolkata, West Bengal, India; ^3^ Department of Pharmacy, Birla Institute of Technology & Science Pilani, Hyderabad, Telangana, India

**Keywords:** phenothiazine-linked, glutamic acid, dendrons, SARS-CoV2

## Abstract

In this report, a structurally unique phenothiazine (PTZ) core is linked with glutamic acid-based dendrons through a solid-phase peptide synthesis approach to access a variety of PTZ-linked dendrons conveniently. Inferior cytotoxicity of anionic surface-linked second-generation glutamic acid-based dendrons would be more desirable for various applications than respective lysine-based dendrons. Solid-phase synthesis of PTZ-linked glutamic acid-based dendrons would be a novel approach to access this class of molecules. These newly synthesized dendrons were screened as an inhibitor against the main protease (M^pro^) enzyme, proposed to be the best target against SARS-CoV-2. The preliminary assay studies designated a moderate response for the M^pro^ inhibition, specifically by tryptophan (Trp)-enriched dendron, among other analogues, which play a vital role in combating COVID-19. Further, the experimental studies realize the essential contribution of the PTZ core in interacting with the M^pro^ enzyme. Molecular dynamics (MD) simulations revealed that the active dendrons formed stable complexes with M^pro^, and the binding affinity of the Trp-based PTZ-linked dendrons was higher than that of the decoy dendron analogue.

## 1. Introduction

Dendrimers are classified as macromolecules consisting of highly branched, well-defined three-dimensional structures. The widespread utility of peptide dendrimers in medical sciences offers several benefits due to their biocompatibility and resistance to proteolytic digestion [1,2]. They have been investigated as antimicrobial, anti-allergic, anti-tumour and antiviral agents [3–5]. Despite easy synthesis and numerous biological applications, the lysine-based dendrimers hold cell cytotoxicity and hematotoxicity disadvantages [6]. In addition, higher-generation dendrimers are realized to be more toxic [6,7], while second-generation (G2) dendrons have been established to bring the best dendritic effect with better biocompatibility over the higher-generation (>G4) dendrimers [7,8]. Enormous efforts have been made to reduce the cytotoxicity of positive dendrimer by modifying it with a negatively charged molecule to reduce the cytotoxicity [9]. Similarly, lysine dendrimer has been modified with negatively charged molecules like sulfonic acid or glutamic acid and their antiviral properties explored [10–12]. Considering this, the second-generation glutamic acid-based dendron could be a better choice than a second-generation lysine-based dendron. In this direction, solid-phase synthesis of varied glutamic acid-based dendrons has been developed recently in our lab [13].

**Figure 1.**
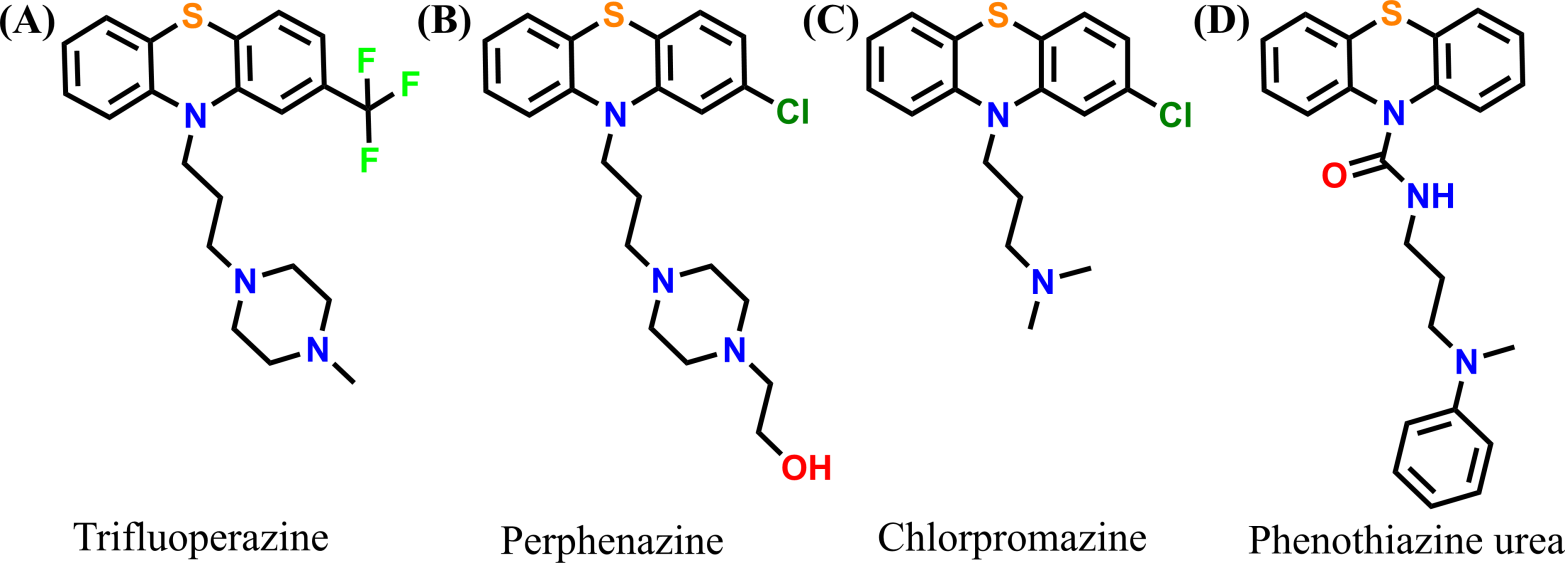
Previously reported SARS-CoV and SARS-CoV-2 inhibitors with phenothiazine core.

Notably, a hydroxyl-PAMAM (polyamidoamine) dendrimer entered a phase II clinical trial against severe COVID-19 [14]. Further, a new coronavirus named SARS-Co-2 caused an outbreak of pulmonary disease in 2019 and spread the infection across the whole world rapidly, causing approximately 7 million deaths [15,16]. Main protease (M^pro^) [17] has been proposed as the best target against SARS-CoV-2 among all the previously studied targets because of its unique cleavage site preference of the peptide bond and significant difference from all the known human proteases [18]. Therefore, inhibiting the SARS-CoV-2 M^pro^ (M^pro^, a vital enzyme responsible for virus replication) can reduce the infection rate by stopping the production of infectious viral particles. Apart from the well-established role of PTZ core as an antipsychotic agent [19], it has also been proven to be vital as a SARS-CoV and SARS-CoV-2 inhibitor. A few relevant PTZ-linked molecules are presented in figure 1, where compounds **A-B** adapt spike protein inhibition [20–22] and compound **C** inhibits through the clathrin-mediated endocytosis process [23]. Also, the potential antiviral role of this core in SARS-CoV-2 through the inhibition of M^pro^ is very well recognized by using the molecule of type **D** [24,25].

In this context, we intended to link the PTZ core with functionalized glutamic acid-based dendron through a solid-state synthesis approach and explored them in the SARS-CoV-2 M^pro^ inhibition screening assay. These preliminary assay studies revealed a moderate response to inhibit the M-protease by tryptophan (Trp)-containing dendrimer. There have been few reports on changes in tryptophan metabolism in COVID-19 patients, indicating its essential role in fighting against COVID-19 [26,27]. We also comprehend the role of this PTZ core by performing molecular docking and MD simulation studies.

## 2. Results and discussion

**Scheme 1.**
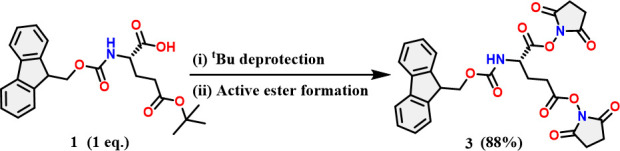
Synthesis of Fmoc-Glu(OSu)-OSu-activated ester. (**i**) 5% TIPS-TFA, 1 h, 25℃. (**ii**) 2.5 eq. EDC.HCl, 2. eq. NHS, 0.02 eq. DMAP, DCM−10℃ to 25℃, 3 h. TIPS = triisopropyl silane, TFA = trifluoroacetic acid, EDC.HCl = *N*-(3-dimethylaminopropyl)-*N*′-ethylcarbodiimide hydrochloride, NHS = *N*-hydroxysuccinimide, DMAP = 4-dimethylaminopyridine.

At the outset, Fmoc-Glu(OSu)-(OSu) (**3**) was synthesized from *N^α^*-Fmoc-l-Glu(O^*t*^Bu)-OH (**1**) as described in Scheme 1. To reach this target **3,** the *tert*-butyl group from *N^α^*-Fmoc-l-Glu(O^*t*^Bu)-OH (**1**) was deprotected to produce the *N^α^*-Fmoc-l-Glu-OH (**2**) and proceeded to the next step without further purification. Compound **2** was activated to afford compound **3** (Scheme 1), which had an excellent yield (88%) and was used directly after water–DCM (dichloromethane) workup without further purification.

### 2.1. Connecting with PTZ core

**Scheme 2.**
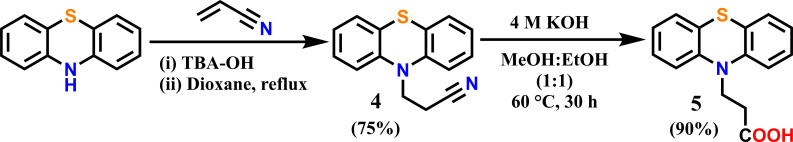
Synthesis of PTZ-CH_2_CH_2_COOH. TBA-OH = tetrabutylammonium hydroxide

The easy functionalization and accessibility of the parent skeleton, PTZ, offer the scope to connect to the dendrimer. The PTZ core was suitably functionalized by adding –CH_2_CH_2_COOH to afford PTZ-CH_2_CH_2_COOH (**5**) through a Michael addition type reaction and followed by hydrolysis (Scheme 2) [28]. This compound **5** can be conveniently connected with the glutamic acid dendrons. Therefore, the molecular PTZ is linked with carboxylic acid functionality (**5**), as shown in Scheme 2.

**Scheme 3.**
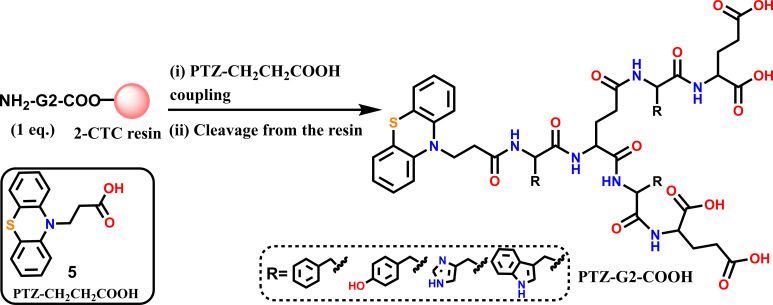
Coupling of **5** on G2 (second-generation) dendron using solid-phase method. (**i**) 1.5 eq. PTZ-CH_2_CH_2_COOH, 1.5 eq. DIC, 1.5 eq. Oxyma, 1.5 eq. DIEA, DMF, 15 min, 50℃; (**ii**) TFA cocktail containing TFA (90%), TIPS (5%) and water (5%) for 2 h under N_2_ atmosphere. DIC = diisopropylcarbodiimide, DIEA = diisopropylethylamine, Oxyma = ethyl cynohydroxyiminoacetate.

Subsequently, functionalized PTZ (**5**) was treated with the 2-chlorotrityl chloride (2-CTC) resin-linked NH_2_-G2-COOH (where G2 is a second-generation dendron) to perform the amide coupling reaction as demonstrated in Scheme 3.

**Figure 2.**
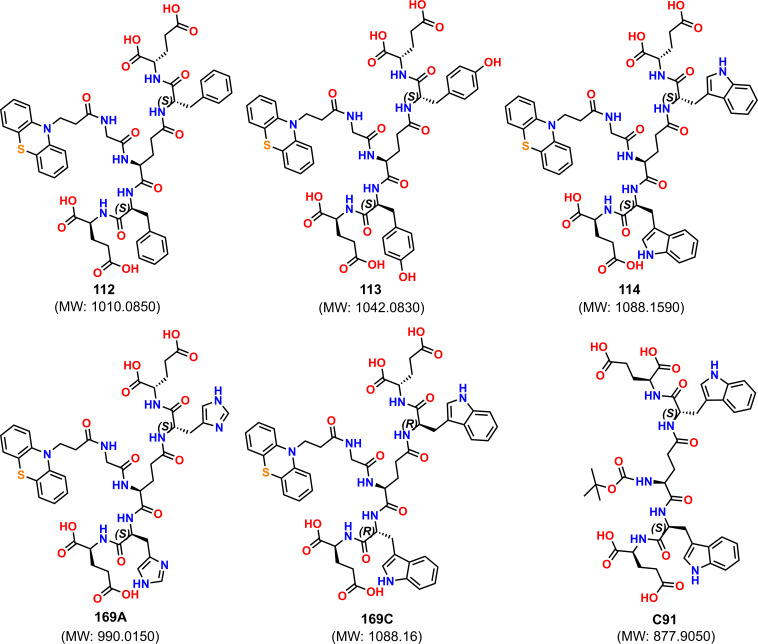
Structural details of synthesized glutamic acid-based dendron, E = glutamic acid (Glu), F = phenylalanine (Phe), H = histidine (His), W = tryptophan, Y = tyrosine (Tyr), and w = d-tryptophan.

Varying the side chain of different amino acids, we could access the following PTZ-linked dendrons. These dendrons (figure 2) were obtained in 17–21% yields in pure state as verified with the HPLC. All of these dendrons are conveniently detected primarily by mass spectrometric studies and also characterized by NMR spectroscopy, wherever possible. All of these dendrons are soluble in pH 7 buffer and DMSO as well since these dendrons contain glutamic acid, which has p*K*a values below 4.3 (glutamic acid p*K*a*_1(COOH)_* = 2.19, p*K*a*_2(side chain COOH)_* = 4.25).

## 3. SARS-CoV-2 main protease inhibitor screening assay

**Figure 3.**
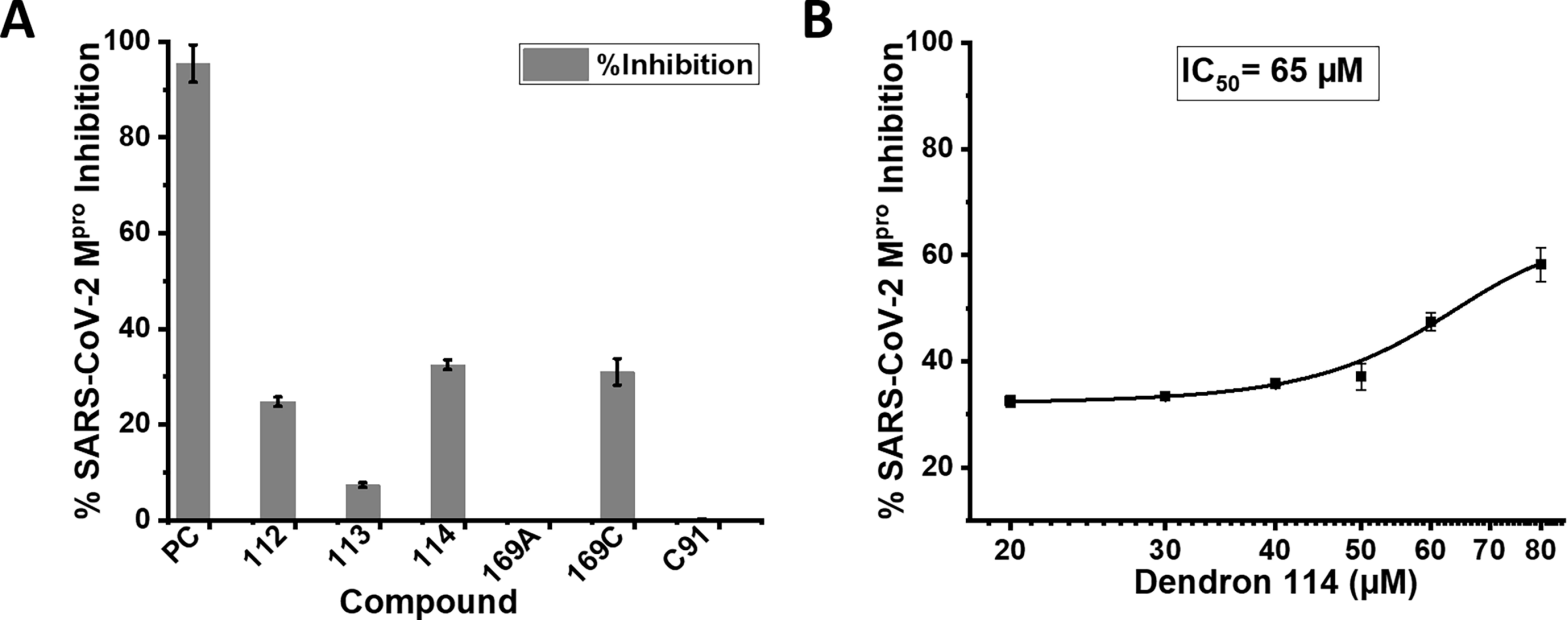
(A) Screening of SARS-CoV-2 M^pro^ inhibition assay with glutamic acid-based PTZ dendrons, where the concentration of all dendrons, including decoy **C91,** was taken at 20 μM and positive control (PC) 500 μM. (B) SARS-CoV-2 M^pro^ inhibition study with compound **114** for IC_50_ calculation. Positive control (PC) = SARS-CoV-2 main protease inhibitor (GC376). The concentrations of the compounds are chosen based on the IC_50_ value of the positive control (0.15 ± 0.03 μM) [30]. Except for compounds **113**, **169A** and **C91,** all other compounds, like 112 (25% inhibition), 114 (33% inhibition) and **169C** (31% inhibition), have shown ≥25% inhibition.

An initial screening was performed with these dendrons using a SARS-CoV-2 main protease assay (limited availability), shared by Cayman Chemicals (Catalogue Number: 701960) [29]. After performing the assay protocol using dendrons Ptz−**112**, **113**, **114**, **169A** and **169C** and analysis (see experimental details), the percent inhibition or percent activity for each inhibitor was determined as shown in figure 3A. The best performance was exhibited by l- and d-tryptophan-containing dendrons **114** and **169C** compared with the positive control (PC) SARS-CoV-2 main protease inhibitor (GC376) [30]. The phenylalanine-linked dendron showed a comparative inhibition, but tyrosine and histidine-connected dendrons were completely non-responsive against SARS-CoV-2 M^pro^. There was another histidine-connected isomeric analogue **169B** (see details in electronic supplementary material), which was not further screened.

Next, the determined IC_50_ (the concentration that shows 50% inhibition) of tryptophan-linked dendron **114** appeared as 65 μM (figure 3B). To define the role of the PTZ core, the tryptophan analogue without PTZ, i.e. compound **C91**, was tested under identical conditions. The non-responsiveness of **C91** against SARS-CoV-2 main protease validates the significant role of the PTZ core in this inhibition study. Further, molecular docking studies were performed to explain the role of PTZ and tryptophan residues of these dendrons with SARS-CoV-2 M^pro^.

## 4. Dendron-M^pro^ docking

The optimized structure of SARS-CoV-2 M^pro^ was validated prior to molecular docking, and the results of Ramachandran plot analysis revealed that 92.6% of the residues were within the most favoured regions, while only 0.4% of the residues were within the disallowed areas, indicating that the quality of the model was good. Further estimation of the model quality with ProSA-web demonstrated that the quality of the prepared structure was comparable to that of structures of similar size, determined with NMR, in the PDB.

The docking protocol was validated by redocking the co-crystallized peptide-like inhibitor, GC376, in the active site of SARS-CoV-2 M^pro^. The RMSD value between the docked and crystal poses was determined to be 0.802 Å, which indicated that the docking algorithm could effectively predict the native binding pose of the co-crystallized ligand (electronic supplementary material, Figure A). The docking scores of the five dendrons and the **C91** decoy dendron are provided in table 1. The results indicated that the docking scores and ligand efficiency values of the active dendrons, namely, **112**, **114** and **169C**, were superior to those of the inactive dendrons, **113** and **169A**, as well as the **C91** decoy dendron. The docking scores of the top five scoring poses of each dendron are provided in the electronic supplementary material, Table 1. The stability of the dendron-M^pro^ complexes was further determined by 100 ns MD simulations.

**Table 1.** Comparison of the docking scores of the five dendrons and the **C91** decoy.

dendron/decoy		docking scores	ligand efficiency
active	**112**	−9.044	−0.126
	**114**	−8.352	−0.107
	**169C**	−10.219	−0.131
inactive	**113**	−3.656	−0.049
	**169A**	−3.092	−0.044
decoy	**C91**	−3.353	−0.050

### 4.1. Trajectory analysis for the assessment of complex stability

**Figure 4.**
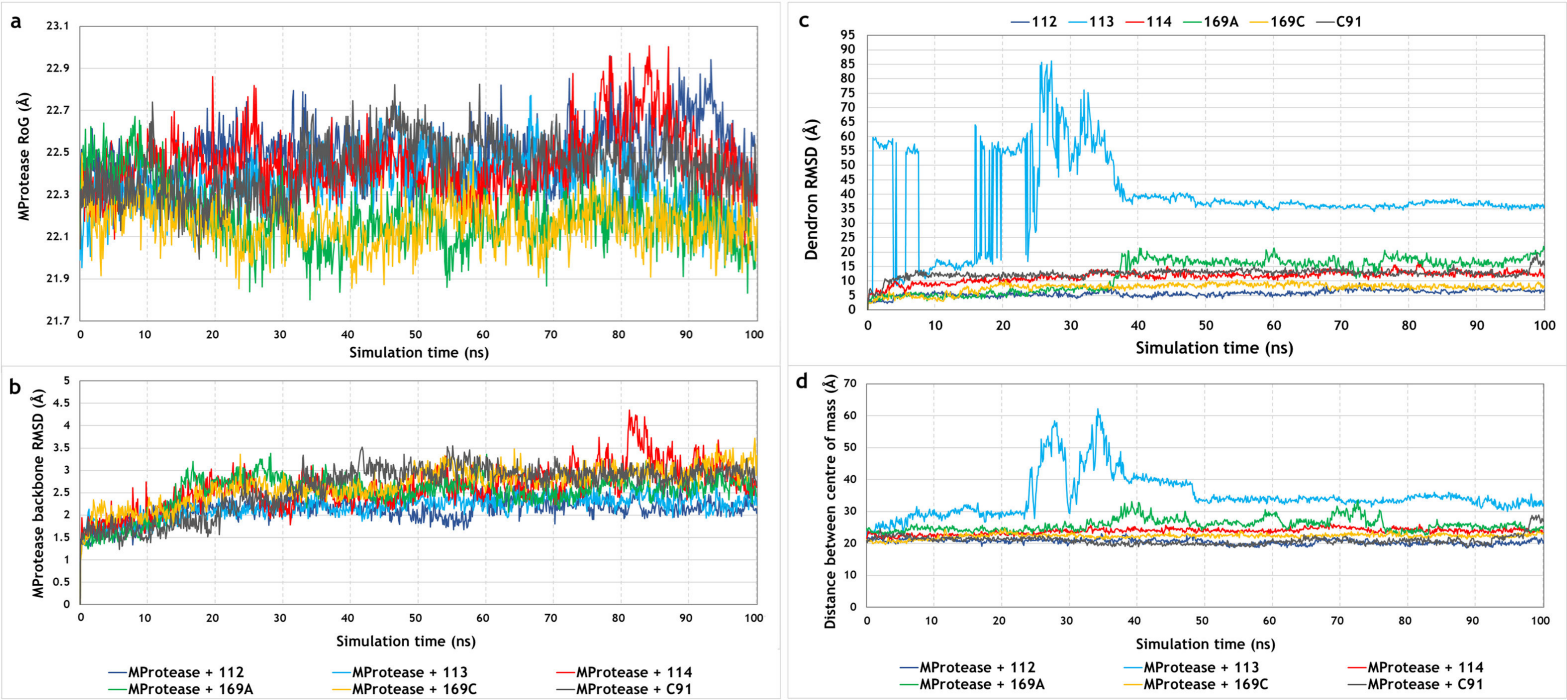
Fluctuations in the (a) RoG and (b) backbone RMSD values of SARS-CoV-2 M^pro^ bound to the active, inactive and decoy dendrons. (c) Fluctuations in the RMSD values of the dendrons over the 100 ns trajectory. (d) Distances between the centres of mass of M^pro^ and the dendrons over the 100 ns production run.

Analysis of the 100 ns MD trajectories revealed that the radius of gyration (RoG) and backbone RMSD values of SARS-CoV-2 M^pro^ remained steady throughout the 100 ns production run, implying that dendron binding did not induce any structural alterations in the protein and that the protein fold remained intact after dendron binding (figure 4a,b). The analysis of the simulation trajectories revealed that the active dendrons, namely, **112**, **114** and **169C**, remained bound to the active site of SARS-CoV-2 M^pro^ throughout the entire 100 ns production run and did not dislodge from the catalytic site at any time point, which aligned with their steady values of RMSD (figure 4c). Complex stability was further corroborated by measuring the distances between the centres of mass of M^pro^ and the active dendrons that remained steady throughout the 100 ns simulation trajectory (figure 4d). However, the inactive dendrons, namely, **113** and **169A**, did not form stable complexes with M^pro^. It was observed that dendron **113** shifted out of the binding site towards the beginning of the trajectory and completely dissociated from the complex at around 24 ns. However, it re-associated with M^pro^ at around 40 ns and remained very superficially bound at a site distant from the active catalytic site of M^pro^ until the end of the production run. These observations corroborated the RMSD values of dendron **113** and the distances between the centres of mass of M^pro^ and dendron **113** throughout the 100 ns trajectory (figure 4c,d). Although dendron **169A** did not dissociate from the complex, it completely shifted out of the binding site at around 38 ns and remained very superficially associated with the complex until the end of the production run, which correlated with the fluctuations in the RMSD values and distances between the centres of mass (figure 4c,d). The **C91** decoy dendron also shifted out of the active site but remained tightly associated at a site distant from the active site of M^pro^ throughout the trajectory (figure 4c,d).

### 4.2. Dendron-M^pro^ interaction stability

The analysis of the dendron-M^pro^ interaction frequencies demonstrated that dendron **112** formed highly stable hydrogen bonds with His 164 and Glu 166 of occupancies 74% and 97%, respectively, and a highly stable π–π interaction of occupancy 84% with His 163. The high occupancy values indicated that these interactions were critical for the stability of the **112**-M^pro^ complex (table 2**,** electronic supplementary material, Figure B). Dendron **112** also formed hydrogen bonds with His 41 and Asn 142 as well as water bridges with Cys 145, Glu 166 and Asp 187, and the lower occupancy of these interactions implied that they played a less prominent role in complex formation. As expected, dendrons **113** and **169A** did not form any interactions with M^pro^, which was attributed to their dissociation from the complex during the simulation. The PTZ moiety of dendron **114** formed hydrophobic interactions with Met 49 of occupancy 54%, indicating the role of the PTZ moiety in the formation of the **114**-M^pro^ complex. Dendron **114** also formed water bridges with Thr 190 and Gln 192 of occupancies 61% and 54%, respectively, of which the occupancy of the interaction with Thr 190 increased to 71% in the last 20 ns of the run. Dendron **169C** formed a highly stable hydrogen bond of occupancy 82% with Thr 190, which was critical for complex stability. It also formed a hydrogen bond of occupancy 69% with Glu 166 and a weaker water bridge with Gln 189 of occupancy 36% (table 2**,** electronic supplementary material**,** Figure B). The **C91** dendron formed stable hydrogen bonds of occupancies 63% and 88% with Pro 168. **C91** did not form any interactions with the active site of M^pro^ due to its shift from the active site.

**Table 2.** Occupancy of the dendron–M^pro^ interactions throughout 100 ns.

dendron	nature of interaction	residue	occupancy over 100 ns (%)
**112**	π–π interaction	His 163	83
		His 172	33
	water bridge	Cys 145	30
		Cys 145	30
		Glu 166	42
		Asp 187	32
	hydrogen bond	His 41	45
			47
		Asn 142	52
		His 164	97
		Glu 166	74
**113**	no interactions with M^pro^	—	—
**114**	hydrophobic interaction	Met 49	54
	hydrogen bond	Gln 189	31
	water bridge	Thr 190	61
		Gln 192	54
**169A**	no interactions with M^pro^	—	—
**169C**	π–π interaction	His 41	39
	hydrogen bond	Thr 190	82
		Glu 166	69
	water bridge	Gln 189	36
**C91**	hydrogen bond	Pro 168	63
			88

## 5. MM-GBSA analyses

The analysis of the Molecular Mechanics with Generalized Born and Surface Area solvation (MM-GBSA) energies over the simulation trajectories revealed that the binding affinities of the active dendrons, **112**, **114** and **169C**, were higher than those of the inactive dendrons, namely, **113** and **169A**, and the decoy **C91** dendron (table 3), as indicated by the more negative values of MM-GBSA energy for the active dendrons. The high binding affinity of **112**, **114** and **169C** was primarily attributed to contributions from the van der Waals energy term, followed by the lipophilic energy term. The binding affinity of dendron **112** was highest among the active dendrons and was likely attributed to the added contribution from the Coulomb energy term and the higher contribution of the van der Waals energy term.

**Table 3.** Comparison of the MM-GBSA energy of the dendrons with M^pro^, averaged over the 100 ns simulation trajectory.

complex	MMGBSA energy	coulomb energy	covalent binding energy	lipophilic energy	generalized Born electrostatic solvation energy	van der Waals energy
**M** ^**pro**^ **+112**	−57.578	−7.491	5.126	−14.311	18.703	−51.603
**M** ^**pro**^ **+113**	−22.225	−12.736	1.984	−5.248	18.827	−22.622
**M** ^**pro**^ **+114**	−47.995	6.639	0.526	−18.491	15.886	−49.755
**M** ^**pro**^ **+169A**	−34.612	55.556	2.372	−11.404	−41.405	−35.805
**M** ^**pro**^ **+169C**	−49.289	22.067	3.269	−17.541	−1.445	−51.654
**M** ^**pro**^ **+C91**	−29.408	55.839	3.416	−9.991	−41.184	−35.698

## 6. Conclusions

In summary, a few important glutamic acid-based dendrons were conveniently linked with PTZ core using a newly developed solid-phase synthetic protocol. The initial screening of these dendrons using a SARS-CoV-2 main protease assay indicated a decent inhibition ability with 65 μM IC_50_, only with the tryptophan-linked dendron analogue. However, both Trp and PTZ units were necessary to exhibit such activity through strong hydrophobic interactions, resulting in a stable complex formation with the associated receptor. The results of MD simulations revealed that the active dendrons, **112**, **114** and **169C**, formed stable complexes with M^pro^, while the inactive and decoy dendrons dissociated from the active site. MM-GBSA analysis further revealed that the binding affinities of the active dendrons for M^pro^ were higher than those of the inactive and decoy dendrons, which corroborated with the experimental findings. This study will gain considerable importance to pave a path for the PTZ-linked glutamic acid dendrons as a new class of molecules to exhibit various antiviral activities.

## 7. Experimental section

**Materials and reagents:** *N*-(3-Dimethylaminopropyl)-*N′*-ethylcarbodiimide hydrochloride (EDC.HCl), *N*-Hydroxysuccinimide (NHS), HPLC grade Trifluoroacetic acid (TFA), *N*, *N*′-Diisopropylcarbodiimide (DIC), *N*, *N*-Diisopropylethylamine (DIEA), *N*, *N*-Dimethylformamide (DMF) and Dichloromethane (DCM) were purchased from Sisco Research Laboratories (SRL), India. 4-(Dimethylamino)pyridine was obtained from Sigma-Aldrich, India. 2-Chlorotrityl chloride (2-CTC) resin was purchased from Supra Sciences, India. HPLC-grade acetonitrile (CH_3_CN) was procured from Thermo Fisher Scientific, India. Guanidinium chloride (Gu.HCl), ethyl cynohydroxyiminoacetate (Oxyma), triisopropylsilane (TIPS), all the natural and non-natural *N^α^*-Fmoc protected amino acids (AA) along with side-chain protecting as Glu(O^*t*^Bu), Thr(^*t*^Bu), Trp(Boc) were purchased from Chem-Impex International, USA.

### 7.1. Reverse-phase HPLC and LC-MS analysis

Analytical reverse-phase (RP) HPLC was performed on an Agilent 1260 Infinity II HPLC using a reverse phase-silica-bound column Agilent Zorbax 300SB-C3 (5 μm), 4.6 mm × 150 mm at a flow rate of 0.9 ml min^−1^ with a linear gradient of 10–64% buffer B in buffer A over 9 min or 10–100% buffer B in buffer A over 15 min at 40°C (buffer A = 0.1% TFA in H_2_O; buffer B = 0.08% TFA in acetonitrile), unless otherwise stated. The UV absorbance of column eluent was monitored at 214 nm or 280 nm wavelength. The high-resolution ESI mass spectra were obtained on an Agilent 1290 Infinity II/6530 Q-TOF LC-MS (capillary voltage 4000 V, gas temperature 325°C, nebulizer pressure 50 psi, drying gas flow 11 l min^−1^, fragmentor voltage 200 V, skimmer voltage 60 V). The mass of the compounds was calculated based on the most abundant isotopologue predicted by Agilent Isotope Distribution Calculator (v. 7.0.7024.0) software unless otherwise stated. Agilent MassHunter Qualitative Analysis (v. B.07.00) software was used for the analysis and reported the most abundant isotopologue observed. Purification of dendrons was done with a reverse phase Waters 1525 preparative HPLC system using Waters Xselect peptide CSH C18 (5 μm, 130 Å, 10 x 250 mm) using an appropriate shallow gradient of linearly increasing concentration of buffer D in buffer C (buffer C = 0.1% AcOH in H_2_O; buffer D = 0.08% AcOH in acetonitrile) at a flow rate of 5 ml min^−1^ at 50°C. ESI-MS identified fractions containing the purified target dendrons, and selected pure fractions were then pooled and lyophilized.

#### 7.1.1. General protocol for machine-assisted Fmoc solid-phase peptide synthesis

Fmoc-solid-phase peptide synthesis (SPPS) was performed by following the reported protocol [31] with minor modifications except for activated *N^α^*-Fmoc-l-Glu(OSu)-OSu ester (**3**) coupling. All the dendrons were synthesized using an automated peptide synthesizer (Tribute-UV/IR from Protein Technologies, USA). Each amino acid was coupled on the 2-Cl-(Trt)-Cl resin for 15 min at 50°C under an N_2_ atmosphere using amino acids (0.25 M), DIC (0.25 M) as a coupling reagent and Oxyma (0.25 M) with N,N-diisopropylethylamine (DIEA) (0.025 M) as additives, unless otherwise mentioned. After every AA coupling cycle, the Fmoc group was deprotected using 20% piperidine treatment at 50°C. After the synthesis, the resin was dried by washing with DCM (3×) and diethyl ether (2×) and used for global deprotection. The global deprotection of the dendrons was performed using TFA (90%), TIPS (5%) and water (5%) as a cleavage cocktail followed by the TFA evaporation under N_2_ flow inside a well-ventilated fume hood. The cleaved dendrons were precipitated and washed with cold diethyl ether 3–4 times. Dry crude dendrons were dissolved in 6 M guanidinium chloride and loaded directly onto a preparative HPLC column for purification.

##### 7.1.1.1. Synthesis of 3

5 g (12 mmol) of *N^α^*-Fmoc-l-Glu(O^t^Bu)-OH **1** was treated with 20 ml of 5% TIPS in TFA for 60 min at RT, followed by the TFA removal *in vacuo*. Crude *N^α^*-Fmoc-l-Glu-OH **2** was used for the next step without any purification. 4.4 g (12 mmol) of *N^α^*-Fmoc-l-Glu-OH, 3.4 g (25.2 mmol) of NHS, 5.8 gm (30 mmol) of EDC.HCl and 29 mg (0.24 mmol) of DMAP were taken together in 100 ml RB and kept at −10°C, followed by adding DCM. The mixture was stirred overnight by increasing the temperature slowly to RT. After the completion of the reaction, an acidic water–DCM (3×) workup was done, followed by sodium bicarbonate–DCM (3×) workup and brine (1×) wash to remove the by-product and excess reagent. The DCM layer was dried over anhydrous MgSO₄ and concentrated to minimum volume followed by the addition of cold diethyl ether to get a white precipitate and wash with cold diethyl ether (2×). The organic phase was dried *in vacuo* to get 6 gm (10.6 mmol; 88% yield) of **3** as a white solid, which was used in the next step directly. The chemical structure of the final product was confirmed by ^1^H (electronic supplementary material, Figure S11A) and ^13^C (electronic supplementary material, Figure S11B) NMR spectroscopy. **^1^H-NMR** [300 MHz (DMSO-*d_6_*, 25°C)]: *δ* 8.18 (br, 1H; NH), 7.89 (br, 2H; CH), 7.71 (m, 2H; CH), 7.42 – 7.33 (m, 4H; CH), 4.63 (m, 1H; CH), 4.37 (m, 2H; CH_2_), 4.27 (m, 1H; CH), 2.94 (m, 2H; CH_2_), 2.82 (m, 8H; CH_2_), 2.19 (m, 2H; CH_2_). **^13^C-NMR** [75.5 MHz (DMSO-*d_6_*, 25°C)]: *δ* 170.2, 168.2, 155.9, 143.8, 143.65, 140.8, 127.7, 127.1, 125.2, 120.17, 66.0, 64.9, 51.1, 46.6, 26.7, 25.8, 25.5.

##### 7.1.1.2. Synthesis of activated 3-(10H*-*phenothiazin-10-yl) propanoic acid (5)

Compound **5** was synthesized following the previously reported method with slight modification [28]. Tetrabutylammonium hydroxide (1.5 ml, 25% in MeOH) was added to an ice-cooled stirring solution of phenothiazine (4 g, 20.0 mmol) and acrylonitrile (15 ml), and the exothermic reaction was allowed to warm to room temperature. After the reaction subsided, dry dioxane (30 ml) was added. The reaction mixture was kept under reflux for 3 h. The mixture was precipitated by pouring it into vigorously stirred distilled water and recrystallized from cold acetone. An off-white solid of **4** was obtained (4.53 g, 18.0 mmol, yield 90%). ESI-MS *m/z* calculated for [C_15_H_12_N_2_SNa]^+^ = 275.0614 Da; found = 275.0627 Da. The chemical structure of the final product was confirmed by ^1^H (electronic supplementary material, Figure S12A) and ^13^C (electronic supplementary material, Figure S12B) NMR spectroscopy. **^1^H-NMR** [300 MHz (DMSO-*d_6_*, 25°C)]: *δ* 7.26–7.21 (br, 4H; CH), 7.09–6.97 (br, 4H; CH), 4.22 (m, 2H; CH_2_), 2.92 (m, 2H; CH_2_). **^13^C-NMR** [75.5 MHz (DMSO-*d_6_*, 25°C)]: *δ* 143.9, 127.8, 127.4, 124.3, 123.1, 118.8, 116.0, 42.3, 16.0.

Further, compound **4** (2 g) was suspended in 110 ml of MeOH:EtOH (1:1) followed by the addition of 18 ml of 4 N KOH solution. The reaction was kept at 60°C for 30 h. After reaction completion, the mixture was cooled down to room temperature, followed by the pH adjustment to 3 with 2 N HCl. The precipitate was filtered out and washed with water having pH 3, followed by drying in the oven overnight, resulting in 1.8 gm (6.6 mmol, yield 84%) grey colour precipitate. ESI-MS *m/z* calculated for [C_15_H_13_NO_2_S]^+^ = 271.0667 Da; found = 271.0686 Da. The compound was used directly for the next step without any further purification.

## 8. General protocol for the G2 dendron synthesis

The G2 dendron was synthesized by machine-assisted SPPS at 50°C (see **General protocol** for machine-assisted Fmoc synthesis). Stepwise synthesis of dendron was carried out on 2-CTC resin (substitution = 0.6 mmol g^–1^) with a 0.05 mmol scale. The coupling of activated *N^α^*-Fmoc-l-Glu(OSu)-OSu ester was done at 50°C for 15 min by taking 0.5 equivalent in DMF (0.11 M) in an automated peptide synthesizer with continuous stirring under N_2_ atmosphere. Dendron was cleaved from the resin and analysed by LC-MS on an analytical scale.

### 8.1. Coupling of 3-(10H*-*phenothiazin-10-yl) propanoic acid (2) on G2 dendron

2.5 equivalent of 3-(10H*-*phenothiazin-10-yl) propanoic acid (**2**) (0.4 M), Oxyma (0.4 M), DIEA (0.04 M) was dissolved in DMF followed by the addition of N,N'-diisopropylcarbodiimide (DIC) (0.4 M) and added over the resin containing **G2** dendron. PTZ-coupled dendron was cleaved from the resin (see **General protocol** for machine-assisted Fmoc synthesis for the cleavage protocol) and purified through preparative RP-HPLC (electronic supplementary material, Figure S2). The pure fraction was pooled and lyophilized.

#### 8.1.1. Synthesis of G2 dendron Ptz-GE(FE-COOH)FE-COOH (112)

The 0.05 mmol G2 dendron GE(FE-COOH)FE-COOH was synthesized and analysed (electronic supplementary material, Figure S1) as mentioned above (see **General protocol for the G2 dendrons synthesis**). Observed mass [M+H]^1+^ (**ESI-MS**): 757.3117 ± 0.01 Da (most abundant isotopologue), calculated mass [M+H]^1+^: 757.3039 Da (most abundant isotopologue).

Dendron Ptz-GE(FE-COOH)FE-COOH (**112**) was synthesized and purified (figure 2) as mentioned above (see **General protocol for the G2 dendrons synthesis**). The pure fraction was pooled and lyophilized to get 11 mg (0.011 mmol, 22%) off-white powder with 99.9% purity. Observed mass [M+H]^1+^ (**ESI-MS**): 1010.3501 Da (most abundant isotopologue), calculated mass [M+H]^1+^: 1010.3600 Da (most abundant isotopologue). The chemical structure of the final product was confirmed by ^1^H (electronic supplementary material, Figure S13A) and ^13^C (electronic supplementary material, Figure S13B) NMR spectroscopy. **^1^H-NMR** [300 MHz (DMSO-*d_6_*, 25°C)]: *δ* 12.46 (br, 4; COOH), 8.57–7.98 (br, 6; NH (amide)), 7.31–6.95 (br, 18; CH), 4.55 (br, 2; CH_2_), 4.27–4.08 (br, 5; CH), 3.68 (br, 2; CH_2_), 3.09–3.05 (br, 2; CH_2_), 2.82–2.63 (br, 4; CH_2_), 2.35 (br, 4; CH_2_), 2.01–1.88 (br, 6; CH_2_), 1.64 (br, 2; CH_2_). **^13^C-NMR** [75.5 MHz (DMSO-*d_6_*, 25°C)]: *δ* 173.8, 173.7, 173.1, 173.0, 172.8, 172.4, 171.8, 171.1, 170.5, 168.4, 144.3, 137.9, 137.7, 129.3, 129.2, 128.1, 127.7, 127.1, 126.3, 123.0, 122.6, 115.5, 54.2, 51.5, 43.1, 42.0, 37.5, 37.2, 33.0, 31.4, 30.1, 30.0, 28.5, 26.3, 26.2.

#### 8.1.2. Synthesis of G2 dendron Ptz-GE(YE-OH)YE-OH (113)

The G2 dendron GE(YE-COOH)YE-COOH was synthesized on a 0.05 mmol scale and analyzed (electronic supplementary material, Figure S3) as mentioned above (see **General protocol for the G2 dendrons synthesis**). Observed mass [M+H]^1+^ (**ESI-MS**): 789.2966 Da (most abundant isotopologue), calculated mass [M+H]^1+^: 789.2937 Da (most abundant isotopologue).

The dendron Ptz-GE(YE-COOH)YE-COOH (**113**) was synthesized and purified as described above (see **General protocol for the G2 dendrons synthesis**) and yielded 10 mg (0.01 mmol, 20%) of white powder with 99.5% purity (electronic supplementary material, Figure S4). Observed mass [M+H]^1+^ (**ESI-MS**): 1042.3419 Da (most abundant isotopologue), calculated mass [M+H]^1+^: 1042.3499 Da (most abundant isotopologue). The chemical structure of the final product was confirmed by ^1^H (electronic supplementary material, Figure S14A) and ^13^C (electronic supplementary material, Figure S14B) NMR spectroscopy. **^1^H-NMR** [300 MHz (DMSO-*d_6_*, 25°C)]: *δ* 12.45 (br, 4; COOH), 9.15 (br, 2; OH), 8.54–7.95 (m, 6; NH (amide)), 7.20–6.94 (br, 12; CH), 6.65 (br, 4; CH), 4.45 (br, 2; CH_2_), 4.26–4.08 (br, 5; CH), 3.75–3.61 (br, 3; CH_2_), 2.92 (br, 2; CH_2_), 2.70–2.62 (br, 3; CH_2_), 2.35 (br, 4; CH_2_), 2.00–1.68 (br, 8; CH_2_). **^13^C-NMR** [75.5 MHz (DMSO-*d_6_*, 25°C)]: *δ* 173.8, 173.7, 173.2, 173.1, 173.0, 172.9, 171.7, 171.1, 170.5, 168.2, 155.8, 144.3, 130.3, 130.2, 128.0, 127.8, 127.7, 127.1, 123.0, 122.6, 115.5, 115.0, 54.8, 54.7, 51.6, 51.4, 43.1, 42.0, 36.7, 36.4, 32.9, 30.2, 30.0, 28.7, 26.2.

#### 8.1.3. Synthesis of G2 dendron Ptz-GE(WE-COOH)WE-COOH (114)

The G2 dendron GE(WE-COOH)WE-COOH was synthesized on 0.1 mmol scales and analysed (electronic supplementary material, Figure S5) as mentioned above (see **General protocol for the G2 dendrons synthesis**). Observed mass [M+H]^1+^ (**ESI-MS**): 835.3297 ± 0.001 Da (most abundant isotopologue), calculated mass [M+H]^1+^: 835.3257 Da (most abundant isotopologue).

The rest half (0.05 mmol) amount of resin containing GE(WE-COOH)WE-COOH was utilized for the synthesis of dendron Ptz-GE(WE-COOH)WE-COOH (**114**) and purified as described above (see **General protocol for the G2 dendrons synthesis**) to get 20 mg (0.018 mmol, 18%) of off-white powder with 99.9% purity (electronic supplementary material, Figure S6). Observed mass [M+H]^1+^ (**ESI-MS**): 1088.3853 Da (most abundant isotopologue), calculated mass [M+H]^1+^: 1088.3818 Da (most abundant isotopologue). The chemical structure of the final product was confirmed by ^1^H (electronic supplementary material, Figure S15A) and ^13^C (electronic supplementary material, Figure S15B) NMR spectroscopy. **^1^H-NMR** [300 MHz (DMSO-*d_6_*, 25°C)]: *δ* 12.42 (br, 4; COOH), 10.79 (s, 2; NH (Indole ring)), 8.64–7.88 (m, 6; NH (amide)), 7.78–7.62 (m, 2; CH), 7.31–6.94 (m, 16; CH), 4.59–4.56 (br, 2; CH), 4.33–4.08 (br, 5; CH), 3.70–3.65 (br, 2; CH_2_), 3.21–3.16 (br, 2; CH_2_), 3.02–2.91 (br, 2; CH_2_), 2.61 (br, 2; CH_2_), 2.38–2.32 (br, 4; CH_2_), 1.98–1.89 (br, 6; CH_2_), 1.68 (br, 2; CH_2_). **^13^C-NMR** [75.5 MHz (DMSO-*d_6_*, 25°C)]: *δ* 173.9, 173.8, 173.4, 173.3, 173.2, 173.1, 172.4, 171.7, 171.5, 171.1, 170.9, 170.7, 170.5, 168.9, 168.3, 144.4, 136.2, 136.1, 127.8, 127.4, 127.2, 127.1, 124.4, 124.2, 124.0, 123.7, 123.1, 123.0, 122.6, 120.9, 118.8, 118.6, 118.2, 115.5, 111.4, 110.2, 110.0, 109.9, 109.8, 53.8, 53.6, 52.5, 51.7, 51.5, 43.1, 42.0, 33.0, 31.5, 30.3, 30.1, 30.0, 27.9, 27.3, 26.3.

#### 8.1.4. Synthesis of G2 dendron Ptz-GE(HE-COOH)HE-COOH (169A)

The 0.05 mmol of dendron Ptz-GE(HE-COOH)HE-COOH (**169A**) was synthesized as described above (see **General protocol for the G2 dendrons synthesis**). *N^α^*-Fmoc-l-His-OH (0.25 M) was coupled for 10 min at RT followed by heating at 50°C for 5 min using DIC (0.25 M) as a coupling reagent, and Oxyma (0.25 M) with DIEA (0.025 M) as additives. Dendron Ptz-GE(HE-COOH)HE-COOH (**169A**) was cleaved from the resin (see **General protocol** for machine-assisted Fmoc synthesis for the cleavage protocol) and purified through preparative RP-HPLC (figure 8). The pure fraction was pooled out and lyophilized to get 9 mg (0.01 mmol, 18%) of white powder with 99.8% purity. Observed mass [M+H]^1+^ (**ESI-MS**): 990.3408 Da (most abundant isotopologue), calculated mass [M+H]^1+^: 990.3410 Da (most abundant isotopologue). The chemical structure of the final product was confirmed by ^1^H (electronic supplementary material, Figure S16A) and ^13^C (electronic supplementary material, Figure S16B) NMR spectroscopy. **^1^H-NMR** [300 MHz (DMSO-*d_6_*, 25°C)]: *δ* 8.41–7.81 (m, 8; NH), 7.19–6.94 (m, 12; CH), 4.45 (br, 2; CH_2_), 4.16–4.09 (br, 5; CH), 3.75 (s, 2; CH_2_), 2.93 (br, 4; CH_2_), 2.64 (br, 2; CH_2_), 2.27–1.80 (m, 12; CH_2_). **^13^C-NMR** [75.5 MHz (DMSO-*d_6_*, 25°C)]: *δ* 174.1, 174.0, 173.8, 173.5, 171.6, 171.4, 171.0, 170.6, 168.7, 144.3, 134.6, 134.4, 127.7, 127.1, 123.0, 122.6, 117.2, 115.5, 52.9, 52.2, 51.9, 43.0, 42.0, 33.0, 30.3, 30.1, 28.7, 26.7, 26.5.

#### 8.1.5. Synthesis of G2 dendron Ptz-GE(hE-COOH)hE-COOH (169B)

The dendron Ptz-GE(hE-COOH)hE-COOH (**169B**) was synthesized in 0.01 mmol scale and purified by RP-HPLC as described above (same as compound **169A**) to get 1.7 mg (0.002 mmol, 17%) white powder with 99.8% purity (electronic supplementary material, Figure S9). Observed mass [M+H]^1+^ (**ESI-MS**): 990.3408 Da (most abundant isotopologue), calculated mass [M+H]^1+^: 990.3410 Da (most abundant isotopologue).

#### 8.1.6. Synthesis of G2 dendron Ptz-GE(wE-COOH)wE-COOH (169C)

The 0.01 mmol of dendron Ptz-GE(wE-COOH)wE-COOH (**169C**) was synthesized and purified by preparative RP-HPLC as described above (see **General protocol for the G2 dendrons synthesis**), yielding 2 mg (0.002 mmol, 19%) of off-white powder with 98% purity (electronic supplementary material, Figure S7). Observed mass [M+H]^1+^ (**ESI-MS**): 1088.3818 Da (most abundant isotopologue), calculated mass [M+H]^1+^: 1088.3818 Da (most abundant isotopologue).

#### 8.1.7. Synthesis of G2 dendron Boc-E(WE-COOH)WE-COOH (C91)

The 20 mg (0.03 mmol) of G2 dendron E(WE-COOH)WE-COOH (0.05 mmol) was treated with NaHCO_3_ (0.2 mmol) followed by the addition of (Boc)_2_O (0.1 mmol) in 2 ml MeCN:H_2_O (4:1) at RT for 24 h to protect the amine group. The mixture was neutralized with 0.5M HCl and lyophilized after the completion of the reaction. The lyophilized crude was dissolved in 6 M guanidinium chloride and purified through preparative RP-HPLC to get 16 mg (0.018 mmol, 60%) yield of (**C91**) as white fluffy solid with 99% purity (electronic supplementary material, Figure S10). Observed mass [M+H]^1+^ (**ESI-MS**): 878.3622 Da (most abundant isotopologue), calculated mass [M+H]^1+^: 878.3567 Da (most abundant isotopologue). The chemical structure of the final product was confirmed by ^1^H (electronic supplementary material, Figure S17A) and ^13^C (electronic supplementary material, Figure S17B) NMR spectroscopy. **^1^H-NMR** [300 MHz (DMSO-*d_6_*, 25°C)]: *δ* 12.43 (br, 4; COOH), 10.79 (s, 2; NH (Indole ring)), 8.58-8.30 (m, 4; NH (amide)), 7.75-7.66 (m, 2; CH), 7.31-6.97 (m, 8; CH), 6.75-6.73 (br, 1; NH), 4.58 (br, 2; CH), 4.32 (br, 2; CH), 3.74 (br, 1; CH), 3.19-2.86 (m, 5; CH_2_), 2.37-2.35 (br, 4; CH_2_), 2.06-1.95 (br, 5; CH_2_), 1.64 (br, 2; CH_2_), 1.33-1.05 (br, 9; CH_3_). **^13^C-NMR** [75.5 MHz (DMSO-*d_6_*, 25°C)]: *δ* 173.8, 173.2, 173.1, 172.8, 172.1, 171.8, 136.1, 127.2, 124.1, 120.9, 120.8, 118.7, 118.2, 111.3, 111.2, 110.0, 109.7, 78.0, 53.5, 51.6, 30.2, 30.1, 28.2, 27.8, 26.3, 21.1.

## 9. SARS-CoV-2 main protease inhibitor screening assay protocol

### 9.1. SARS-CoV-2 main protease inhibitor screening assay company name: Cayman chemicals

Assay protocol: Step I: Add 70 µl SARS-CoV-2 Main Protease Assay Buffer and 10 µl of solvent (1% DMSO) (the same solvent concentration used to dissolve the unknown inhibitor and the positive control SARS-CoV-2 main protease inhibitor (GC376)) to three wells. Mix the contents of the wells by pipetting. Step II: Add 50 µl of SARS-CoV-2 Main Protease Assay Buffer, 20 µl of SARS-CoV-2 Main Protease Enzyme, and 10 µl of solvent to three wells. Use the same solvent concentration used for the unknown inhibitor and the positive control, SARS-CoV-2 Main Protease Inhibitor (GC376). Mix the contents of the wells by pipetting Step-II: Add 50 µl of SARS-CoV-2 Main Protease. Assay Buffer, 20 µl of SARS-CoV-2 Main Protease Enzyme, and 10 µl of unknown inhibitor or the 500 µM positive control, SARS-CoV-2 Main Protease Inhibitor (GC376), working solution to three wells. Mix the contents of the wells by pipetting. Step IV: Incubate for 30 min at room temperature. Step V: Initiate the reactions by adding 20 µl of SARS-CoV-2 Main Protease Substrate to all the wells being used and mix well by pipetting. Step VI: Cover the plate with the 96-Well Cover Sheet (Item No. 400012) and incubate for two hours at room temperature protected from light. Step VII: Remove the plate cover and read the plate with an excitation wavelength of 340 nm and an emission wavelength of 490 nm. It may be necessary to adjust the gain setting to allow for the measurement of all samples.

#### 9.1.1. Analysis: calculation step I

Determine the average fluorescence (AF) of each sample **Step II:** Subtract the AF of the background wells from the AF of the 100% initial activity and inhibitor wells; Step III: Determine the percent inhibition or percent activity for each inhibitor using one of the following equations:


$$\%\text{Inhibition}=\frac{\left(\text{corrected 100}\%\text{ initial activity - corrected inhibitor activity}\right)}{\left(\text{Corrected 100}\%\text{ initial activity}\right)}\times 100$$

$$\%\text{Activity}=\frac{\left(\text{corrected inhibitor activity}\right)}{\left(\text{Corrected 100}\%\text{ initial activity}\right)}\times 100$$

Step IV: Graph the percent inhibition or percent activity as a function of inhibitor concentration to determine the IC_50_ value (the concentration at which there is 50% inhibition) of the inhibitor. Inhibition of recombinant SARS-CoV-2 Main Protease by SARS-CoV-2 Main Protease Inhibitor (GC376).

## 10. M^pro^-dendron docking

The structures of the five dendrons (**112**, **113**, **114**, **169A** and **169C**) and the **C91** decoy dendron were prepared using Maestro, Schrödinger. The three-dimensional structures of the peptide ligands were initially generated with LigPrep, Schrödinger, using the OPLS4 force field [32,33]. The ionization states of the dendrons were kept unaltered during structure generation. The optimal protonation states of the Glu moieties were subsequently generated based on the p*K*a values at pH 7.4, calculated using the PROPKA program in Schrödinger [34]. The structures were finally minimized using the OPLS4 force field, and the minimized structures were used for molecular docking. The M^pro^ of SARS-CoV-2 bound to the peptide-like inhibitor, GC376 (PDB ID: 7D1M), was selected as the receptor for molecular docking [35]. The receptor was prepared using the Protein Preparation Wizard application in Schrödinger [34]. The missing residues and side chains were modelled using Prime [36,37]. The protonation states of the Asn, Gln, Asp and hydroxyl residues were optimized and the non-structural waters were removed. The hydrogen atoms were finally minimized using the OPLS4 force field, while keeping the heavy atoms restrained. The final structure was validated by Ramachandran plot analysis with ProCheck and the model quality was estimated using ProSA-web [38,39].

A cubic grid was prepared around the active catalytic site of M^pro^, based on the binding site of the bound inhibitor, GC376. The coordinates of the centre of the grid were −19.573, 63.460 and 1.033, and the dimensions were 25 Å on all sides. The docking protocol was validated by redocking the co-crystallized inhibitor using the Glide SP-peptide algorithm, and the RMSD between the docked and crystal poses was determined. Generally, an RMSD value ≤2.0 Å indicates a good correlation between the docked and crystal poses. The five PTZ-linked dendrons and the **C91** dendron (without PTZ link) were docked to the active catalytic site of SARS-CoV-2 M^pro^ using the Glide SP-Peptide mode of Schrödinger [40]. The binding poses of the dendrons in the binding site were visualized using Maestro, Schrödinger.

### 10.1. Determination of complex stability by MD simulations

The stability of the docked dendron-M^pro^ complexes was assessed via MD simulations for 100 ns, performed in Desmond [41]. For solvation, the dendron-M^pro^ complexes were placed in an orthorhombic TIP3P water box, and the thickness of the solvent buffer was set to 10 Å. The overall charge was subsequently neutralized by adding the required number of Na^+^ or Cl^−^ ions to the solvated systems, and the final concentration of the solution was adjusted to 0.15 M by adding NaCl. The systems were relaxed using the default protocol in Desmond, which included an initial restrained minimization followed by unrestrained minimization, and four short MD runs. During restrained minimization, the systems were relaxed using Brownian dynamics for 100 ps under the NVT ensemble at a temperature of 10 K, followed by a short MD run for 12 ps under the NVT ensemble at 10 K. The systems were subjected to another MD run for 12 ps under the NPT ensemble at 10 K. During unrestrained minimization, the systems were relaxed by MD simulations for 24 ps using the NPT ensemble. The systems were finally simulated for 100 ns under the NPT ensemble in Desmond, using the OPLS4 force. The temperature was adjusted to 300 K using the Nose-Hoover chain thermostat, and the pressure was maintained at 1.013 bar using the Martyna–Tobias–Klein barostat, with a relaxation time of 2.0 ps. The trajectories were visualized in Desmond and analysed using the Simulation Interactions Diagram tool in Desmond, Schrödinger. The fluctuations in the RMSD and RoG values of M^pro^, the RMSD values of the dendrons and the fluctuations in the distances between the centres of mass of the dendrons and M^pro^ were determined. The occupancy of the dendron-M^pro^ interactions over the 100 ns production run was additionally analysed, and interactions with higher occupancy values were considered to be important for complex formation.

## Data Availability

All the data are available in the ESI [42].
